# Consolidated bioethanol production from olive mill waste: Wood-decay fungi from central Morocco as promising decomposition and fermentation biocatalysts

**DOI:** 10.1016/j.btre.2020.e00541

**Published:** 2020-10-09

**Authors:** Hasna Nait M’Barek, Soukaina Arif, Behnam Taidi, Hassan Hajjaj

**Affiliations:** a Faculty of Sciences of Meknes, Laboratory of Plant Biotechnology and Molecular Biology, BP 11201, Zitoune Meknes City, Morocco; b Cluster of Competency «Agri-food, Safety and Security» IUC VLIR-UOS, Moulay Ismail University, Marjane 2, BP 298, Meknes City, Morocco; c CentraleSupélec, SFR Condorcet FR, CNRS 3417, Paris-Saclay University, European Center of Biotechnology and Bioeconomy (CEBB) – LGPM, 3 Rue des Rouges Terres, 51110, Pomacle, France

**Keywords:** Wood-decay fungi, Biomass, Bioethanol, Fusarium oxysporum, Consolidated bioprocessing

## Abstract

Meknes region is a Moroccan olive-processing area generating high amounts of non-valorized Olive Mill Waste (OMW). Fungi are natural decomposers producing varied enzyme classes and effectively contributing to the carbon cycle. However, structural complexity of biomass and modest performances of wild fungi are major limits for local biorefineries. The objective of current research is to assess the ability of local fungi for bioethanol production from OMW using Consolidated Bioprocessing (CBP). This is done by characterizing lignocellulolytic potential of six wood-decay and compost-inhabiting ascomycetes and selecting potent fermentation biocatalysts. High and diversified activities were expressed by *Fusarium solani* and *Fusarium oxysporum*: 9.36 IU. mL^−1^ and 2.88 IU. mL^−1^ total cellulase activity, 0.54 IU. mL^−1^ and 0.57 IU. mL^−1^ laccase activity, respectively, and 8.43 IU. mL^−1^ lignin peroxidase activity for the latter. *F. oxysporum* had maximum bioethanol production and yield of 2.47 g.L^-1^ and 0.84 g.g^−1^, respectively, qualifying it as an important bio-agent for single-pot local biorefinery.

## Highlights

• First report on lignocellulolytic activity and diversity of fungi from central Morocco.
• Olive Mill Waste (OMW) is a suitable biomass for local biorefinery in Meknes region.
• Fusaria isolates produce high and diversified lignocellulases using Consolidated Bioprocess.
• *Fusarium oxysporum* (76) achieves 2.47 g.L^−1^ bioethanol production and 0.84 g.g^−1^ yield.
• Bioethanol is maximally produced during the oxygen-limiting phase.

## Graphical abstract

**Figure:**
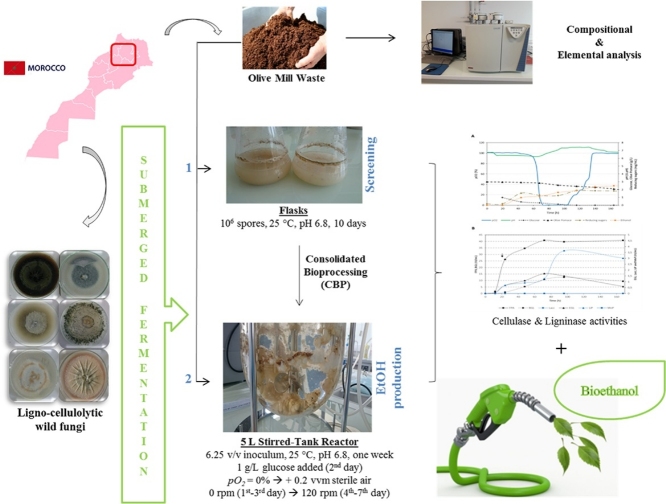

## 1 Introduction

The world’s industrialization rhythm and technological advancements have led to an environmental crisis over the last several decades. The high energy demand, decreasing natural pools of fossil resources, toxic effluents directly rejected in the environment and traditional product design and life cycle (from cradle to grave) are, among others, main factors that made the environmental situation a serious emergency [1,2]. Fortunately and since the declaration of the United Nation’s sustainable development goals in 2015, this issue has become of major concern, and many countries established objectives towards the transition to green energy sources and closing the production loops to reach sustainability. The use of biomass as a renewable energy resource has answered a big part of this issue. Lignocellulose is the most abundant waste on earth and is a common agricultural and food processing by-product directly thrown in nature and non-valorized [3]. Moreover, its biotransformation using microorganisms reduces the competition tension on food-grade resources and enables the production of industrially-valuable enzymes, green biofuels, and a set of highly-wanted platform molecules for bio-based chemistry [4].

To date, several Agri wastes are investigated around the world: corn straw/stover, sugarcane bagasse, and starchy residues as major by-products in Brazil and the United States of America, wheat and rice straw in Asia and countries of the silk road, sorghum straw/stalk in South Africa and finally, wood ships and miscanthus in Europe [[5], [6], [7], [8]]. In north-central Morocco, the region of Meknes is an excellent olive tree region hosting 342 000 ha of olive-growing lands and representing 60 % of national production with 376 tons olives/year. Many processing units are installed (4414 traditional and 344 moderns) and generate huge quantities of olive mill wet-cake in the region [9]. Interestingly, an official and serious declaration for Meknes city in favor of the olive tree was made during the United Nations’ Conference of Parties (COP 22) for climate change, giving a deep commitment to the valorization of its by-products and aiming to derive 30 % of energy needs from their exploitation [10,11].

Though it is an ambitious objective, lignocellulose is a complex matrix and its decomposition in nature implicates microbial consortia with many strains at a time. It is composed of a core of crystalline and amorphous cellulose regions enveloped by hemicellulose and a structurally-diversified polyphenolic polymer, the lignin [12,13]. Besides, olive mill waste is rich in polyphenols and is considered relatively toxic for some microorganisms. Bacteria and fungi are popular natural colonizers of biomass able to produce a pool of different active enzymes (hydrolases, oxidases, and peroxidases) to attack the polymer and retrieve nutrients and energy. In this breakdown process, many enzyme classes are implicated and act in synergy: cellobiohydrolases (CBH) (EC 3.2.1.91), endoglucanases (EG) (EC 3.2.14), β-glucosidases (BGL) (EC 3.2.1.21), laccases (Lacc), lignin peroxidases (LiP), manganese-dependent peroxidases (MnP), lytic polysaccharide monooxygenases (LPMO) and other accessory enzymes (AA) [[14], [15], [16], [17], [18], [19]]. Regarding cellulose hydrolysis, two cellulolytic systems are known in microorganisms, so far: cellulosome and the uncomplexed cellulolytic system [20]. The first is a membrane multi-enzyme compound system found in bacteria from the *Thermocellum* and *Clostridium* genera. The second, a proteomic cocktail of independent extracellular enzymes, is principally characteristic of the fungal kingdom and few bacterial strains such as *Cellulomonas fimi* and *Streptomyces lividans* [21]. The potential of fungi for biomass decomposition is acknowledged through several studies and applications, starting from recognizing the natural potential of wood-decay isolates to the engineering of versatile enzymatic pools for biorefinery. Their strength resides in the diversity of extracellular enzymes they produce and their mechanisms of action: many Carbohydrate-Active enzyme families (CAZymes) that act in synergy and complementary to tear cellulose and hemicellulose into pieces of simple oligosaccharidic chains, the non-selective polysaccharide attack of the LPMOs, lignin mineralization under the action of oxidative, peroxidative and versatile complementary activities, the implication of generated radical cores in the lignin decomposition process and finally, the Fenton chemistry [22,23]. Consequently, the matrix recalcitrance is reduced under efficient fungal treatments and free sugars are progressively released and metabolites produced. Another advantage is that some mold’s cellulases are thermo-tolerant, acid-active, presenting enhanced selectivity due to the presence of a multi-component carbohydrate-binding module (CBM) and sometimes even hosting more than a catalytic domain [24]. Moreover, fermentation of sugars to bioethanol can be operated by selected ascomycetes under specific oxygen conditions considering the advantage that, some species are less sensitive to carbon catabolite repression (CCR), lignin degradation products, and fermentation inhibitors, than others [[25], [26], [27]]. All these factors gathered explain why fungi and their lignocellulolytic cocktails are potent candidates for elevated biomass conversion rates and biofuel productivities.

Bioethanol production is a harsh operational process with an increasing need for potent biocatalysts. Depending on economically-viable scenarios, different lignocellulose sources are being used in the production of 2nd generation bioethanol and their recalcitrance is most of the time encountered by adopting pre-treatment strategies. However, these latter use chemical liquors that generate environmentally toxic effluents (acids, bases, ammonia) or are water- and energy-consuming for heat explosion, for instance [28,29]. Ionic liquids pre-treatments had partially helped to solve this issue with their higher selectivity, lignin removal, and environment preservation. Though, the technique is costly and still under tremendous investigation for use in the industrial scale [30]. Biodegradation using fungal enzymes remains the most effective option to date and fits perfectly into the integrated biorefinery scheme [21]. However, there are still some bottlenecks in the way of building sustainable enzyme-based bioenergy plants with optimized costs. One major challenge that comes before improving growth and production conditions is already the screening of fungal colonizers that can produce interesting lignocellulolytic titers and with less catabolite-repressed or inhibited enzymes [23,31,32]. Manipulation of strains by genome editing tools, protoplast fusion, protein engineering, and other methods were so far rapid routes to enhance strains’ performances but at the same time inflating the charges and not favoring cost-effective processes. Thus, mining highly-active wild fungi from local biotopes remains essential to gain affordable industrial productivities and promote a sustainable local sector. Coming to process design and optimization, a single-pot submerged fermentation (SmF) is favored among all developed bioprocesses in terms of biofuel productivities, integrated scheme, and thus, cost reduction. It relies on a consolidated design where fungal enzymes are *in situ* produced, lignocellulose is degraded and released sugars are simultaneously fermented using the same species [33]. In this sense, some pilot studies have been conducted using white-rot molds from *Trametes* and *Phlebia* genera and succeeded to reach promising ethanol productivities [34,35]. However, basidiomycetes are selective towards substrate characteristics compared to other fungal genera and are not convenient for a majority of Agri waste residues. Moreover, they are known for their low bioethanol tolerance, evaluated to less than 2 % v/v [33]. Ascomycetes of the *Aspergillus*, *Rhizopus*, *Paecylomyces*, *Monilia*, *Neurospora*, *Fusarium*, *Tricoderma,* and *Mucor* genera have been reported as a choice candidate for the consolidated production scheme [27,36,37]. Regarding many metabolic advantages, *Fusarium oxysporum* isolates were specifically experimented for varied biomasses and demonstrated high enzymatic performance, competitive sugar conversion yields, the ability to ferment different biomass-derived pentoses and hexoses and finally, high ethanol tolerance [27,36,38]. The main focus of current research is to evaluate the enzymatic versatility of wild lignocellulolytic fungi isolated in Meknes region (central Morocco) for *in situ* decomposition of OMW as a local biomass residue, and assessing their bioethanol production potential using a CBP strategy.

## 2 Materials and methods

### 2.1 Fungal strains

*Humicola grisea* (61), *Fusarium oxysporum* (76), two *Fusarium solani* species (85 and 102), *Trichoderma atroviride* (88), and *Aspergillus fischeri* (117) were lignocellulolytic fungi from the Biotechnologies and Bio-resources Development Laboratory collection, Moulay Ismail University of Meknes, Morocco (Table 1). They were previously isolated from the same region, validated for cellulase and/or ligninase production in submerged culture, and identified with the Internal Transcribed Spacer (ITS) region of nuclear ribosomal DNA, as reported in our previous work [31]. All strains were maintained in Czapek plates at 4 °C until use.

**Table 1:** Identity and lignocellulolytic profile of fungi used in this study. OMW: olive mill waste, C.A: cellulolytic activity, L.A: lignolytic activity. NA: not available. Lignocellulolytic activities were measured and reported in details in our previous work [31].

Fungi	Substrutum	C.A	L.A	GenBank Accession	Morphology	Fungi	Substrutum	C.A	L.A	GenBank Accession	Morphology
*Humicola grisea*	Wood decay	+	–	NA		*Fusarium solani*	OMW compost	+	+	MK956803	
*Fusarium oxysporum*	Wood decay	+	+	MK956809		*Trichoderma atroviride*	Wood decay	+	–	NA	
*Fusarium solani*	Wood decay	–	+	MK956810		*Aspergillus fischeri*	OMW	+	+	MK956802	

### 2.2 Media and chemicals

Czapek mineral medium was used. Per 1 L MilliQ water: 2 g NaNO_3_, 1 g K_2_HPO_4_, 0.5 g KCl, 0.5 g MgSO_4_*7H_2_O, 0.01 g FeSO_4_*7H_2_O, 1 mL trace metal solution (0.5 g CuSO_4_*5H_2_O, 1 g ZnSO_4_*7H_2_O, in 100 mL MilliQ water) and 3 g carbon source (OMW, cellulose or lignin), pH 6.8. Systematic culture and maintenance of strains were done in solid plates (+16 g.L^−1^ Agar) where 30 g.L^−1^ sucrose was used as the carbon source. Cellulose (∼50 μm particle size, Avicel®) and Lignin Alkali with no reducing sugars (CAS 8068-05-1, reference 471003) were purchased from Sigma Aldrich. All chemicals used were ACS grade, purchased from Sigma Aldrich, or Fischer Scientific, France. Water in all experiments was prepared using Merck Millipore Milli-Q™ Ultrapure Water Purification System (Fischer Scientific, France).

### 2.3 Feedstock origin, preparation, and characterization

Two-phase OMW was sampled from a representative processing company in the Meknes region (Morocco). It was air-dried and ground with SM 300 knife-cutting mill (Ø_sieve_ 1 mm, Retsch GmbH, Germany). Compositional analysis of OMW was done according to standard laboratory protocols of the National Renewable Energy Laboratory (NREL) [[39], [40], [41], [42], [43], [44]] for total solids, ash, extractives, and lignin contents. Carbon, hydrogen, nitrogen, sulfur, and oxygen elements were determined using FLASH 2000 CHNS-O organic elemental analyzer (Thermo Scientific, France; Eager Xperience interface) following the supplier’s protocol. All carbon sources used in fermentation were dried at 40 °C for 24 h before use.

### 2.4 OMW-induced fungal enzymes in SmF

One-week-old culture of each fungus was used to prepare inoculum: 10 mL of Milli-Q water and 20 sterile glass beads (Ø 5 mm, Sigma) were added to the plate and mixed thoroughly to make a spore solution. It was 10 μm aseptically-filtered and checked under microscope (AxioVision, Zeiss, France). Spores were counted using Multisizer 4 Coulter counter (60-μm probe aperture size, Beckman Coulter, France) and their concentration adjusted. 250 mL flasks with 100 mL Czapek-OMW, -cellulose or -lignin was 121 °C sterilized for 20 min, inoculated with 10^6^ spores, and statically incubated at 25 °C for 10 days. Proteins and lignocellulolytic activities were measured after 3, 5, 7, and 10 days according to reference protocols [[45], [46], [47]]. All samples were triplicated.

### 2.5 Bioethanol production

Flaks of Czapek - OMW mineral medium were inoculated with *F. oxysporum* (76) and *F. solani* (102) and statically incubated at 25 °C for 7 days. Culture broths were recovered, centrifuged at 10 000 rpm for 10 min, 0.2 μm aseptically-filtered and supernatants analyzed with High-Performance Liquid Chromatography (HPLC) system for ethanol, sugars, and carboxylic acids produced during fermentation. All samples were triplicated.

*F. oxysporum* (76) was selected for one-week batch fermentation in a bioreactor using OMW as substrate and cellulose as control. 5 L Stirred-Tank Bioreactor (STR) (Global Process Concept BIO, France) was connected to a GX control station and monitored online using C-BIO software. It was inoculated at 6.25 % v/v inoculation ratio from one-week mycelial culture, temperature maintained at 25 °C (online probe control). pH and dissolved oxygen (*pO_2_*) were monitored online using internal Hamilton probes with optical sensing for the latter (VisiFerm DO Arc, Hamilton, Switzerland). *pO_2_* was expressed in terms of % O_2_ partial pressure in the liquid phase of the culture. A command was set to supply 0.2 vvm sterile air when dissolved oxygen is totally consumed (*pO_2_* = 0 %). No stirring was applied for the first three days to enable the start of mycelial growth (low CO_2_ concentration and the absence of shear forces [48]). The stirring speed was after adjusted at 120 rpm using a spiral propeller blade. On the second day of incubation, 1 g.L^−1^ glucose was added to induce the production of fungal cellulases [49]. Samples were aseptically-recovered every day, centrifuged and supernatants analyzed for proteins, lignocellulolytic activities, substrate consumption, reducing sugars, and ethanol.

### 2.6 Analytical methods

**Protein concentration:** Proteins were measured using Pierce Modified Lowry Protein Assay Kit (Thermo Scientifc™, France) in conformance with the manufacturer instructions.

**Cellulase activity:** Cellulases were followed over time in flasks and bioreactor, as three main components: Filter Paper Assay (FPA) for total cellulase activity, CMCase for endoglucanase activity (EG), and cellobiase for β-glucosidase activity (BGL). Standard protocols of the International Union of Pure and Applied Chemistry (IUPAC) [46] were followed to quantify the released reducing sugars using Dinitrosalicylic acid (DNS) modified reagent [50].

**Ligninase activity:** Lignolytic activities were followed over time in flasks and bioreactor as laccase (Lacc), lignin peroxidase (LiP), and manganese-dependent peroxidase (MnP) activities according to [47]. The oxidation of 2,2’-azino-bis(3-ethylbenzothiazolin- 6-sulphonic acid) (ABTS), Veratryl alcohol, and phenol red was followed at 436 nm, 310 nm, and 610 nm, respectively. All enzyme activities were expressed in International Units per Milliliter (IU.mL^−1^), defined as the amount of enzyme capable of catalyzing the transformation of 1 μmole of substrate per minute, under standard protocol conditions. Samples were triplicated and analyzed against enzyme and substrate blanks.

**Substrate consumption:** OMW and cellulose consumptions in bioreactor were determined in time according to [51].

**Reducing sugars:** Reducing sugars produced during substrate decomposition were quantified using the DNS method [50]. 50 mL of growth medium was centrifuged, supernatant mixed with DNS (1/3 v/v), and the mixture boiled for 15 min. Absorbance was read at 540 nm after cooling and appropriate dilution.

**HPLC analysis of fermentation products:** Glucose, glycerol, and carboxylic acids were quantified in flasks using an HPLC system with refractive index detector (HPLC-RI). Ethanol was measured in flasks and bioreactor broths using the same system: Ultimate 3000 apparatus (Fisher Scientific, France) equipped with Aminex HPX-87H HPLC column (9 μm, Bio-Rad, France). The column was heated to 30 °C and eluted with 5 mM H_2_SO_4_ in isocratic mode, flow rate 0.6 mL.min^−1^. Results were visualized and analyzed using Chromeleon 6.8 software.

### 2.7 Data analysis

Results were presented as mean ± standard deviation of triplicate samples. The Least Significant Difference (LSD) was computed using Tukey’s Post-Hoc test under IBM SPSS Statistics 22 software.

## 3 Results

### 3.1 Composition of OMW biomass

OMW analysis resulted in a rich fraction of ethanol-soluble extractives, which constitutes a good non-structural source for the initial growth of fungi. The structure of this biomass is abundant with acid-insoluble lignin (Kalson lignin), up to 37.73 ± 0.65 % (on a Dry Weight basis). Besides, the elemental analysis demonstrated that oxygen content was considerable reaching 35.66 ± 0.71 % (DW), which gives OMW a recalcitrant character. Ash content and the small amount of nitrogen are advantageously interesting for fungal growth. Detailed results of the compositional parameters are summarized in Table 2.

**Table 2:** Compositional and Elemental analysis of OMW. AIL: Acid Insoluble Lignin (Kalson Lignin), ASL: Acid Soluble Lignin. (%) are reported on a Dry Weight (DW) basis. All values are represented as mean ± standard deviation of duplicated samples.

Compositional Analysis						Elemental Analysis (%)				
*Total solids (%)*	*Ash (%)*	*Extractives*		*Lignin content (%)*		*Carbon*	*Hydrogen*	*Nitrogen*	*Sulfur*	*Oxygen*
		*Water soluble (g. g^−1^)*	*Ethanol soluble (%)*	*AIL*	*ASL*					
92,9 ± 0.99	4.91 ± 0.35	0.032 ± 0.002 *glycerol*	20.4 ± 0.3	37.73 ± 0.65	0.000246 ± 0.00009	50,54 ± 0,38	6,44 ± 0,09	2,15 ± 0,06	0,57 ± 0,02	35,66 ± 0,71

### 3.2 Lignocellulolytic fungal cocktails produced in the presence of OMW carbon source

**Fig. 1:**
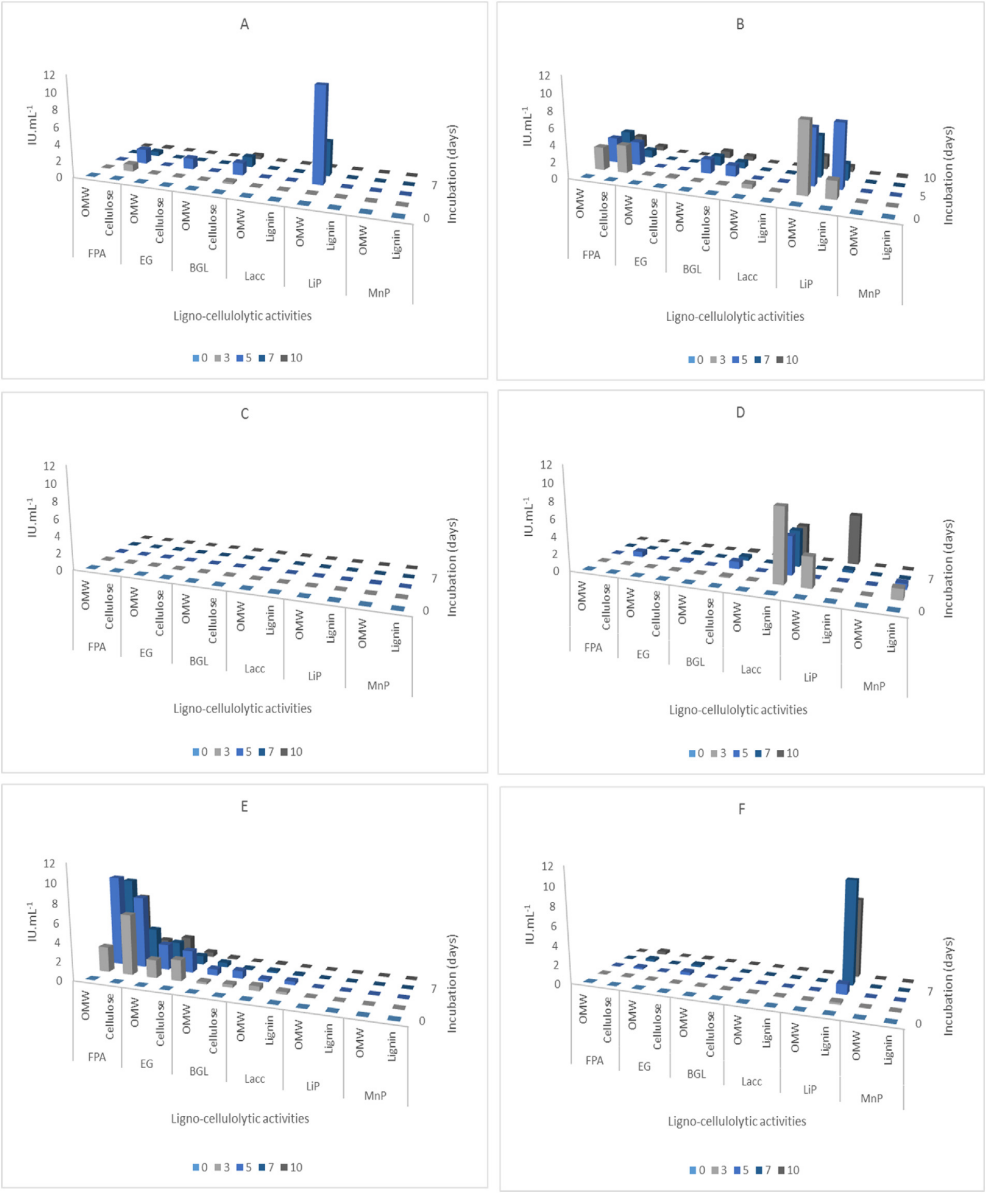
Fungal lignocellulolytic enzymes production in the presence of Olive Mill Waste (OMW) and model substrates (Cellulose, Lignin) at 25 °C for 10 days. **A**: *H. grisea* (61), **B**: *F. oxysporum* (76), **C**: *F. solani* (85), **D**: *T. atroviride* (88), **E**: *F. solani* (102) and **F**: *A. fischeri* (117). FPA: Filter Paper Assay (total cellulase activity), EG: endoglucanase activity, BGL: β-glucosidase activity, Lacc: laccase activity, LiP: lignin peroxidase activity and MnP: manganese-dependent peroxidase activity. IU. mL^−1^: International Unit per Milliliter of supernatant (For interpretation of the references to colour in this figure legend, the reader is referred to the web version of this article.).

Ascomycetes used in this study behaved differently in the presence of OMW and showed a diversified profile of enzymatic cocktails. The most interesting ones were from two Fusaria strains, namely: *F. solani* (102) and *F. oxysporum* (76) (Fig. 1: **E** and **B**). They contained endoglucanases (2.6 and 0.03 IU. mL^−1^), β-glucosidases (0.66 and 1.76 IU. mL^−1^), and a total saccharifying activity reaching 9.36 and 2.88 IU. mL^−1^, respectively. Those cellulolytic activities were maximally expressed either in the 3rd or 5th day of fungal growth. Laccase was also produced by both strains (0.54 and 0.57 IU. mL^−1^, respectively) and LiP only by the latter strain (8.43 IU. mL^−1^). Additionally, *H. grisea* (61) and *T. atroviride* (88) (Fig. 1: **A** and **D**) were interestingly proficient in expressing only LiP activity up to 11 and 3.37 IU. mL^−1^, respectively. It was observed for *H. grisea* (61) and *F. oxysporum* (76) that their cellulolytic systems were preferably induced in the presence of cellulose Avicel® than complex lignocellulosic biomass (total saccharifying activity: 1.74 and 3.31 IU. mL^−1^, respectively). On the other hand, *T. atroviride* (88) and *A. fischeri* (117) (Fig. 1: **D** and **F**) expressed better lignocellulolytic activities in the presence of model substrates. They reached 0.63 and 0.31 IU. mL^-1^ total filter paper activity, 8.5 IU. mL^-1^ laccase, 5.7 and 10.69 IU. mL^-1^ LiP, and 1.2 IU. mL^-1^ MnP activity for both strains, respectively. Results are summarized in Fig. 1 and **Table S1** in the supplementary material.

### 3.3 Bioethanol production by wood-decay and compost-inhabiting fusaria

**Fig. 2:**
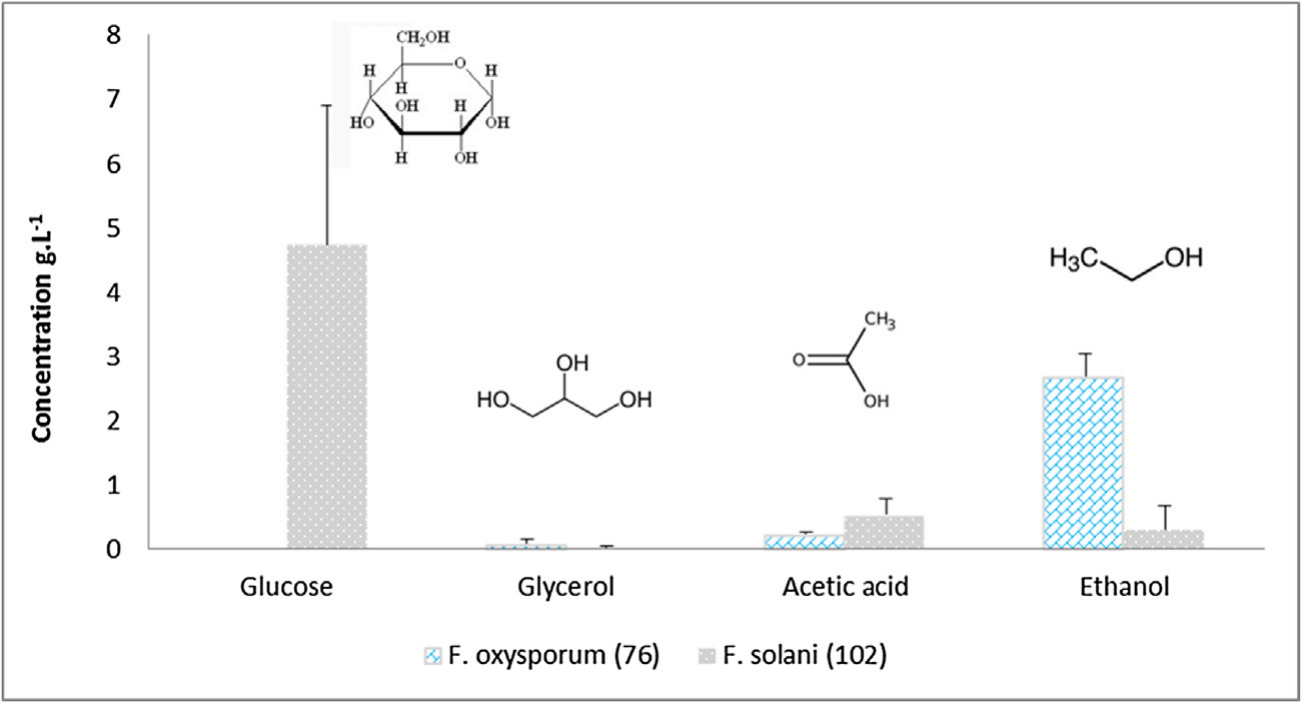
HPLC-RI analysis of glucose, glycerol, acetic acid and ethanol produced after one-week of Olive Mill Waste submerged fermentation using *F. oxysporum* (76) and *F. solani* (102), at 25 °C with no shaking (For interpretation of the references to colour in this figure legend, the reader is referred to the web version of this article.).

After one-week of fungal growth, glucose, glycerol, acetic acid, and ethanol were differently detected in supernatants of *F. oxysporum* and *F. solani* flask cultures (Fig. 2). Glycerol and acetic acid were particularly present in very small concentrations with more acetic acid production by *F. solani* (0.54 g.L^−1^). High glucose level was detected in supernatants of this strain (4.73 g.L^−1^) coupled with a low conversion into ethanol. Besides, *F. oxysporum* produced up to 2.69 g.L^−1^ of ethanol and no sugar was detected in the fermentation broth. The latter was selected for bioethanol production using CBP due to its enzymatic and fermentation interesting patterns.

**Fig. 3:**
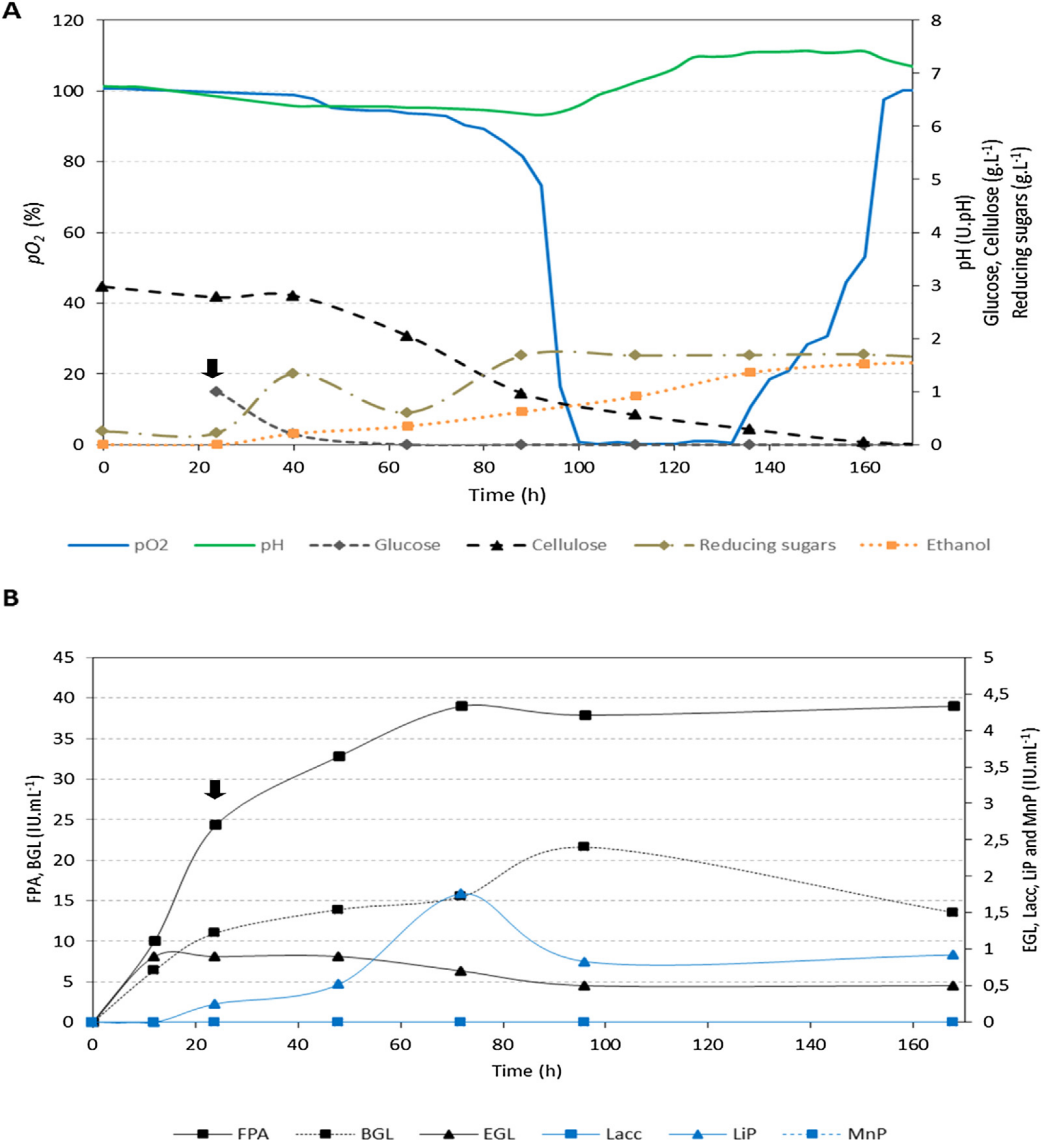
One-week batch fermentation kinetics (**A**) and lignocellulolytic activities (**B**) of *F. oxysporum* (76) in the presence of cellulose at 25 °C and pH 6.8. Air was supplemented when all oxygen was consumed (*pO_2_*_=_ 0 %) in the medium. Supplementation rate was set to 0.2 v.v.m. No agitation was applied for the three first days to enable good start of mycelial growth and it was after that set to 120 rpm for the rest of fermentation. *pO_2_*: partial oxygen pressure in the liquid phase, FPA: Filter Paper Assay for total cellulase activity, EGL: endoglucanase activity, BGL: β-glucosidase activity, Lacc: laccase activity, LiP: lignin peroxidase activity and MnP: manganese dependent peroxidase activity, IU. mL^−1^: International Unit per Milliliter of supernatant. Black small arrow indicates the addition of glucose (For interpretation of the references to colour in this figure legend, the reader is referred to the web version of this article.).

**Fig. 4:**
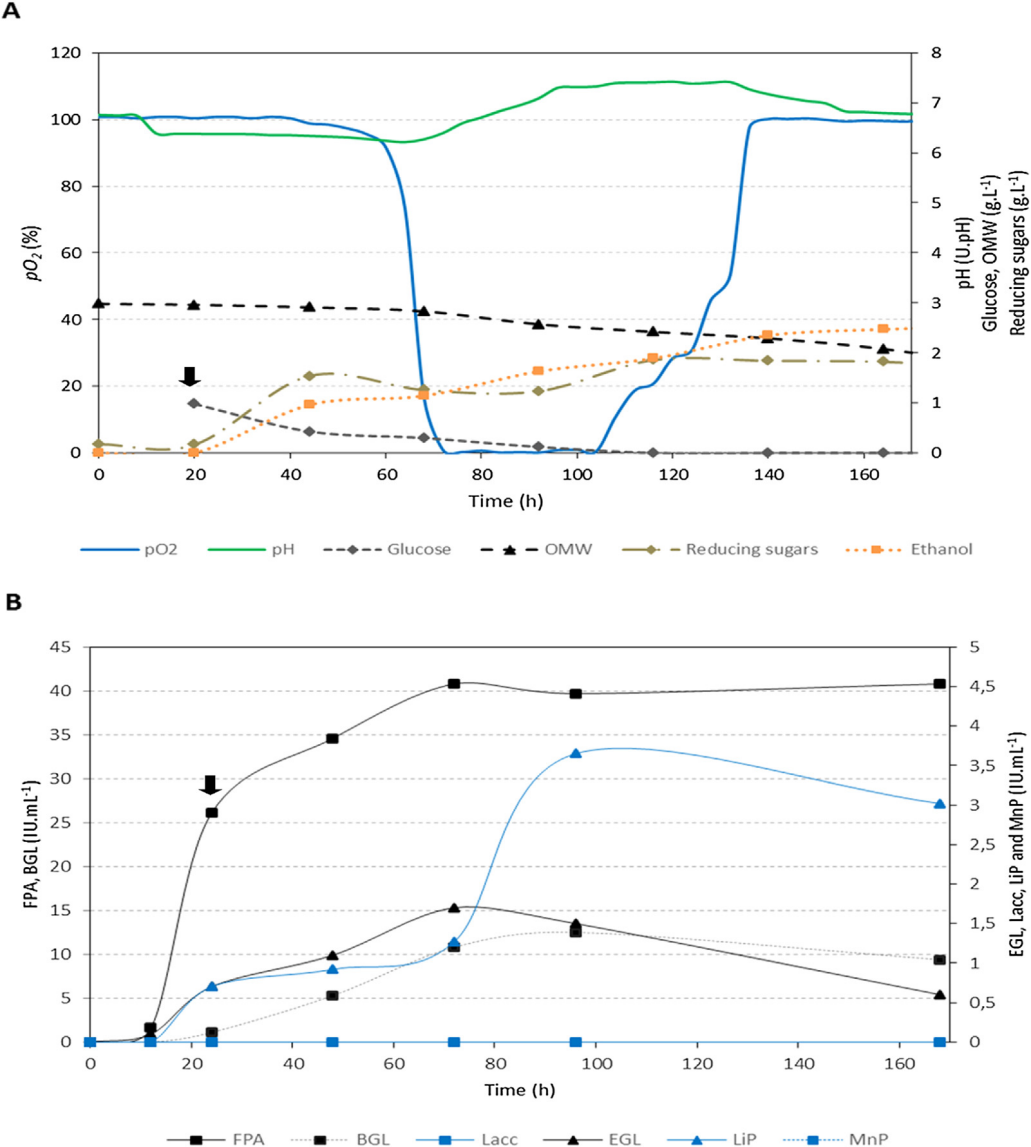
One-week batch fermentation kinetics (**A**) and lignocellulolytic activities (**B**) of *F. oxysporum* (76) in the presence of Olive Mill Waste (OMW) at 25 °C and pH 6.8. Air was supplemented when all oxygen was consumed (*pO_2_*_=_ 0 %) in the medium. Supplementation rate was set to 0.2 v.v.m. No agitation was applied for the three first days to enable good start of mycelial growth and it was after that set to 120 rpm for the rest of fermentation. *pO_2_*: partial oxygen pressure in the liquid phase, FPA: Filter Paper Assay for total cellulase activity, EGL: endoglucanase activity, BGL: β-glucosidase activity, Lacc: laccase activity, LiP: lignin peroxidase activity and MnP: manganese dependent peroxidase activity, IU. mL^−1^: International Unit per Milliliter of supernatant. Black small arrow indicates the addition of glucose (For interpretation of the references to colour in this figure legend, the reader is referred to the web version of this article.).

In batch culture, it was observed during the growth phase of *F. oxysporum* that the fungus started glucose consumption as soon as it was added to the medium. However, different consumption rates were noticed with cellulose or OMW (Table 3). With both carbon sources, glucose addition all along with the selected operational conditions significantly enhanced the expression of lignocellulolytic enzymes: the cellulolytic system of the fungus was fourteen folds higher (Fig. 3, Fig. 4B**, Table S1** in supplementary material). Otherwise, a tardive induction was observed in the expression of lignin peroxidase activity when OMW was used. After 72 h of fermentation, almost all lignocellulolytic enzymes were stable over time indicating the absence of proteases and enzyme inhibitors in the medium. In Fig. 3, saccharification of cellulose started immediately under the action of the produced enzymes, and ethanol was gradually produced reaching a maximum of 1.52 g.L^−1^ at end of fermentation. By 160 h of the batch culture, all cellulose was already hydrolyzed with a consumption rate reaching 45 mg.L^−1^. h^−1^ and high enzyme yields were achieved: total cellulase activity (13 000 IU. g^−1^) and lignin peroxidase activity (586.7 IU. g^−1^) (Table 3). On the other side, OMW was observed to be quite difficult for the strain to decompose and its consumption rate was measured as 13.75 mg.L^−1^. h^-1^ (Table 3, Fig. 4). The substrate was gradually deconstructed with the main participation of hydrolases (total cellulase yield reaching 13 600 IU. g^−1^) and the high and tardive lignin peroxidase activity yielding much more than in the cellulose batch (1 216.7 IU. g^−1^). After one week of growth under selected conditions, 1 g.L^−1^ of OMW was decomposed by *F. oxysporum* with a maximum ethanol production of 2.47 g.L^−1^. Moreover, it was observed that, independently of the carbon source, ethanol production by this *Fusarium* isolate started slow in aerobic conditions and went maximal in the oxygen-limiting phase. Ethanol volumetric productivities reached 18.75 mg.L^−1^. h^−1^ and 40 mg.L^−1^. h^-1^ and yields of 0.51 g.g^−1^ and 0.84 g.g^−1^ in the presence of cellulose and OMW, respectively (Table 3**)**. Besides, oxygen was similarly consumed with an uptake rate of 29.43 μmole.L^−1^. h^−1^ and a maximum consumption was paralleled to the maximum expression of β–glucosidase activity and release of reducing sugar, thus, suggesting the substantial need of oxygen for the transport and catabolism of produced saccharides. Finally, reducing sugars were approximately similarly produced in both cultures with the formation of obviously more fungal biomass in the cellulose batch than the OMW one.

**Table 3:** Fermentation parameters of *Fusarium oxysporum* (76) batch-cultures in the presence of cellulose (C) or Olive Mill Waste (OMW) as sole carbon source, for one week at 25 °C and pH 6.8. Sterile air was supplemented (0.2 v.v.m) when oxygen was totally consumed (*pO2* = 0 %) in the medium. Agitation was set to 120 rpm starting from the 3rd day of growth and till the end of fermentation. Glucose (1 g.L^−1^) was supplemented in the 2nd day of growth for enzyme induction. O_2_: Oxygen, G: Glucose, FPA: total cellulase activity, EG: endoglucanase activity, BGL: β-glucosidase activity, LiP: lignin peroxidase activity.

Carbon source	Consumption Rates			Ligno-cellulolytic enzymes								Ethanol production		
				Volumetric Productivity (IU.L^−1^. h^−1^)				Yield (IU. g^−1^)				Final concentration (g.L^−1^)	Volumetric Productivity (mg.L^−1^. h^−1^)	Yield (g. g^−1^)
	O_2_ (μmole.L^−1^. h^−1^)	C / OMW (mg.L^−1^. h^−1^)	G (mg.L^−1^. h^−1^)	FPA	EG	BGL	LiP	FPA	EG	BGL	LiP			
**C**	29,43	45	50	1191,7	75	533,3	51,7	13000	300	7200	586,7	1,52	18,75	0,51
**OMW**	29,43	13,75	28,33	2041.7	50	229,2	0,1	13600	566,7	4166,7	1216,7	2,47	40	0,84

## 4 Discussion

Biomass decomposition by fungi is a complex process implicating the production of a large set of cellulase and ligninase classes acting in ultimate synergy. Several wild molds produce secretomes rich in these enzymes, but because of their natural living mode (most of the time in consortia), their performances remain modest with an enzymatic pool poorly diversified. This trait is also related to the fungal genomic code of life and its environmental adaptation across time [22]. In the present study, we observed that the cellulolytic system of *H. grisea* (61) was significantly induced in the presence of pure cellulose in the medium. It was well diversified and rich in β-glucosidase activity, which is mostly a limiting factor in a single-pot saccharification and fermentation process of lignocellulose. This wood-decay fungus isolated in Morocco was more enzymatically versatile compared to other soil-inhabiting isolates [52], suggesting the importance of the original substratum, geographical location, and environmental conditions in the mining of industrially-valuable molds. Additionally, lignin peroxidase activity was higher when OMW was used, than what was reported [53]. Its kinetic of expression along with the low and late endoglucanase activity supports the fact that, even though mechanically pretreated, OMW remains a complex and recalcitrant matrix for the enzymatic deconstruction by this strain. The use of steam explosion pretreatment was recommended prior to biodegradation in order to enhance saccharification yields by *H. grisea* [54,55].

To the best of our knowledge, *Trichoderma* and *Aspergillus* strains were never reported for the high production of ligninolytic enzymes, and actual industrial ligninases are mainly produced using basidiomycetes such as *Phanerochaete chrysosporium, Pleurotus ostreatus, Lentinula edodes*, *Trametes versicolor*, *Ganoderma lucidum* and others [[56], [57], [58]]*. T. atroviride* and *A. fischeri* studied here are fortunately strong agents of lignin mineralization, characterized by high, stable, and early-produced enzymatic titers. As these biocatalysts are crucial for industrial operations such as textile desizing, paper, and pulp bleaching, detergent and animal feed formulations, bioenergy production, and others [45,46], to date in biorefinery, for instance, *T. reesei* and *A. nidulans* are selected via genome-engineering of natural parent isolates to achieve highly cellulolytic potential and coop with the limited original system. Despite this enabled large-scale production of versatile and process resistant CAZymes, engineering steps induced additional time and excessive charges [[47], [48], [49]].

Since optimization of biofuel production costs is essential for a viable and sustainable sector, the race has lastly risen for the valorization of locally-abundant biomasses and the mining of highly biomass-induced fungal strains [59,60]. In this perspective, OMW is key biomass in our context and *Fusarium* genus holds the most interesting patterns from this study. *Fusaria* represent a clade of versatile biological agents, highly sporulating and very adapted to stressful environmental variations. This explains why a huge number are known serious plant pathogens causing severe agricultural damage. *F. oxysporum* (76) and *F. solani* (102) here-studied were high saccharifying agents of OMW with the production of a mix of remarkable endoglucanases and β–glucosidases at a time. Additionally, the cellulolytic system of the former was fourteen folds improved using the selected operational conditions in CBP, and ethanol yield was equal to the fermentation of simple monosaccharides [61]. Glucose uptake and ethanol production in flasks experiments revealed a difference between both strains. It was clear the presence of channeling or metabolic bottlenecks in *F. solani* (102) limiting the use of glucose and more ethanol production. It was demonstrated that *F. oxysporum* is among very few CBP-adapted fungi that under oxygen-limiting conditions, decompose lignocellulose and ferment the generated sugars into ethanol with yields as similar as for simple glucose-xylose fermentation [[61], [62], [63], [64]] [This study]. Additionally, its potential to metabolize crystalline cellulose under simultaneous saccharification and fermentation confirmed results from previous works with more prominent ethanol yields as here-presented [65]. An important cultural parameter behind reaching those yields is the use of nitrate as a nitrogen source. Panagiotou G. and coworkers [61,62,65] demonstrated that nitrate utilization by a cumin isolate from Greece enabled the activation of a special denitrification system in *F. oxysporum* composed of three NADH-dependent enzymes: nitrate reductase, nitrite reductase, and nitric oxide reductase and a good regeneration of NAD^+^ was then achieved. The importance of this fact resides in the role this cofactor plays in converting hemicellulosic pentoses (xylose and arabinose) into D-xylulose and then in integrating the Pentose Phosphate Pathway (PPP) [61,62]. When grown on L-arabinose alone, less fungal mycelium is formed and high levels of Glucose 6-Phosphate (G6P) are measured in this fungus, which is related to the lack in arabinose transport into the cell and to G6P channeling difficulties to the PPP [62]. Taking the above-mentioned cultural and metabolic factors into consideration, we understand that under oxygen-limiting conditions, *F. oxysporum* (76) principally uses glucose and xylose issued from biomass break-down for ethanol production and arabinose does not significantly contribute to the process. Finally, these findings highlight the importance of this wood-decay wild fungus for high ethanol production from local OMW and suggest its convenience for use in consolidated and integrated bioethanol production.

## 5 Conclusion

The use of OMW as local biomass is an important factor towards developing sustainable biorefineries in the region of Meknes. Moreover, the availability of potent wild lignocellulolytic ascomycetes that can break it down and convert it into bioethanol is with no doubt advantageous. Despite the matrix recalcitrance, the strain’s enzymatic diversity and potential are able enough to initiate a lignin attack, penetrate the pores, reach cellulose microfibrils, and finally, enchain the decomposition and fermentation processes. Besides, highly-active enzymes produced by these strains constitute valuable ingredients for industrial cocktails enrichment. *Fusarium oxysporum* was shown to be a perfect saccharification and fermentation biocatalyst with great potential for use in consolidated bioethanol production from OMW.

## CRediT authorship contribution statement

**Hasna Nait M’Barek:** Conceptualization, Data curation, Formal analysis, Investigation, Methodology, Visualization, Writing - original draft, Writing - review & editing. **Soukaina Arif:** Data curation, Formal analysis, Writing - original draft, Writing - review & editing. **Behnam Taidi:** Data curation, Formal analysis, Methodology, Resources, Writing - review & editing. **Hassan Hajjaj:** Conceptualization, Funding acquisition, Investigation, Project administration, Resources, Validation, Writing - review & editing.

## Declaration of Competing Interest

The authors report no declarations of interest.
