# Supplementary material for: Consolidated bioethanol production from olive mill waste: Wood-decay fungi from central Morocco as promising decomposition and fermentation biocatalysts
Source: Biotechnol Rep (Amst). 2020 Oct 9;28:e00541. doi: 10.1016/j.btre.2020.e00541 (PMC7578684; doi:10.1016/j.btre.2020.e00541)
Supplement: Supplementary file 1 [file mmc1.docx]

**Table S1:** Maximum expression of ligno-cellulolytic activities in the presence of Olive Mill Waste (OMW) and cellulose or lignin as model substrates. 61 (A): *Humicola grisea*, 76 (B): *Fusarium oxysporum*, 85 (C): *Fusarium solani*, 88 (D): *Trichoderma atroviride*, 102 (E): *Fusarium solani* and 117 (F): *Aspergillus fischeri*. FPA: Filter Paper Assay for total cellulase activity, EG: Endoglucanase activity, BGL: β-Glucosidase activity, Lacc: Laccase activity, LiP: Lignin Peroxidase activity and MnP: Manganese dependent Peroxidase activity. Day of maximum activity is indicated between brackets. nd: not detected. IU.mL^-1^: International Unit per Milliliter of supernatant. Values followed by different upper letters are significantly different (p < 0.05).

| **Activities** | **Cellulase (IU.mL^-1^)** | | | **Ligninase (IU.mL^-1^)** | | |
| --- | --- | --- | --- | --- | --- | --- |
|  | **FPA** | **EG** | **BGL** | **Lacc** | **LiP** | **MnP** |
| **Fungus** | **OMW** | | | | | |
| **61 (A)** | nd | 0,017 ± 0,006^b^  *(10)* | nd | nd | 10,998 ± 0,5^a^  *(5)* | nd |
| **76 (B)** | 2,876 ± 0,22^b^  *(5)* | 0,0355 ± 0,008^b^  *(3)* | 1,76 ± 0,1^a^  *(5)* | 0,5705 ± 0,071^a^  *(3)* | 8,4305 ± 0,13^b^  *(3)* | nd |
| **85 (C)** | 0,024 ± 0,03^c^  *(5)* | 0,036 ± 0,006^b^  *(5)* | nd | nd | 0,004 ± 0,003^d^  *(5)* | 0,032 ± 0,004^a^  *(5)* |
| **88 (D)** | nd | 0,005 ± 0,001^b^  *(5,7)* | nd | nd | 3,37 ± 0,12^c^  *(3)* | nd |
| **102 (E)** | 9,3655 ± 0,06^a^  *(5)* | 2,6035 ± 0,09^a^  *(5)* | 0,66 ± 0,017^b^  *(5)* | 0,54 ± 0,071^a^  *(3)* | nd | nd |
| **117 (F)** | nd | nd | nd | nd | nd | nd |
| **Fungus** | **Cellulose / Lignin** | | | | | |
| **61 (A)** | 1,74 ± 0,09^c^  *(5)* | 1,3 ± 0,16^b^  *(5)* | 1,46 ± 0,12^a^  *(5)* | nd | nd | nd |
| **76 (B)** | 3,31 ± 0,15^b^  *(3)* | 0,14 ± 0,02^d^  *(3)* | 1,28 ± 0,09^a^  *(5)* | nd | 7,56 ± 0,08^b^  *(5)* | 0,097 ± 0,003^b^  *(10)* |
| **85 (C)** | 0,027 ± 0,01^e^  *(7)* | 0,005 ± 0,001^e^  *(7)* | nd | nd | 0,002 ± 0,001^d^  *(10)* | 0,01 ± 0,002^c^  *(3)* |
| **88 (D)** | 0,63 ± 0,1^d^  *(5)* | 0,19 ± 0,02^d^  *(5)* | 0,84 ± 0,1^b^  *(5)* | 8,5 ± 0,12^a^  *(3)* | 5,73 ± 0,7^c^  *(10)* | 1,21 ± 0,15^a^  *(3)* |
| **102 (E)** | 7,46 ± 0,54^a^  *(5)* | 2,29 ± 0,21^a^  *(5)* | 0,79 ± 0,13^b^  *(5)* | 0,36 ± 0,03^b^  *(5)* | nd | nd |
| **117 (F)** | 0,31 ± 0,04^d^  *(10)* | 0,31 ± 0,07^c^  *(5)* | nd | nd | 10,7 ± 0,23^a^  *(7)* | nd |
